# Joint Learning of Emotion and Singing Style for Enhanced Music Style Understanding

**DOI:** 10.3390/s25247575

**Published:** 2025-12-13

**Authors:** Yuwen Chen, Jing Mao, Rui-Feng Wang

**Affiliations:** 1 School of Humanities and Arts, Hunan Institute of Traffic Engineering, Hengyang 421219, China; chenyuwen2025@hnjt.edu.cn; 2 Department of Crop and Soil Sciences, College of Agriculture and Environmental Sciences, University of Georgia, Tifton, GA 31793, USA

**Keywords:** music singing style, music emotion, AI4Music, multi-task learning

## Abstract

Understanding music styles is essential for music information retrieval, personalized recommendation, and AI-assisted content creation. However, existing work typically addresses tasks such as emotion classification and singing style classification independently, thereby neglecting the intrinsic relationships between them. In this study, we introduce a multi-task learning framework that jointly models these two tasks to enable explicit knowledge sharing and mutual enhancement. Our results indicate that joint optimization consistently outperforms single-task counterparts, demonstrating the value of leveraging inter-task correlations for more robust singing style analysis. To assess the generality and adaptability of the proposed framework, we evaluate it across various backbone architectures, including Transformer, TextCNN, and BERT, and observe stable performance improvements in all cases. Experiments on a benchmark dataset, which were self-constructed and collected through professional recording devices, further show that the framework not only achieves the best accuracy on both tasks on our dataset under a singer-wise split, but also yields interpretable insights into the interplay between emotional expression and stylistic characteristics in vocal performance.

## 1. Introduction

Music style is a fundamental factor in shaping listeners’ perception of musical works and in defining the individuality of vocal performance [1,2]. A thorough understanding of music style is important for music information retrieval tasks, including indexing, classification, and the retrieval of large-scale music collections. It is also critical for personalized recommendation systems and AI-assisted content creation, where subtle stylistic cues influence processes of generation and adaptation. In addition to these practical applications, computational study of musical style holds theoretical importance because it provides a structured perspective for examining how technical vocal attributes interact with affective expressivity, thereby offering insight into how musical meaning is shaped and communicated.

Despite its conceptual and applied significance, computational modeling of musical style remains a challenging and relatively underdeveloped research area. One of the central difficulties lies in the inherently multifaceted nature of vocal expression [3,4], which involves both stylistic techniques (for example, Pop or Bel canto singing) and the nuanced conveyance of emotional states (for example, joy or sadness). These intertwined dimensions make it difficult to isolate, represent, and interpret stylistic features, and they present persistent challenges for current data-driven modeling approaches.

Earlier research on music style classification predominantly relied on handcrafted acoustic features, such as timbre, pitch, and MFCCs, in combination with shallow classification models. Although these methods achieved a certain level of performance, they exhibit two notable limitations. First, handcrafted features often fail to capture the semantic depth and contextual dependencies embedded in vocal signals, particularly when stylistic information is conveyed through subtle temporal variations [5,6,7,8,9]. Second, shallow models have limited capacity to integrate heterogeneous cues across different representational levels, from low-level acoustic characteristics to high-level semantic structures.

In recent years, deep learning methods have achieved notable success across a wide range of domains, inspiring research on more expressive and data-driven approaches to music analysis [10,11,12,13]. Recent studies have attempted to address these issues by employing deep learning architectures [14,15,16,17,18], including convolutional and recurrent neural networks, to learn discriminative vocal representations directly from raw signals or time–frequency representations. However, the majority of these works adopt a single-task learning paradigm, treating emotion recognition and singing style classification as independent objectives. This separation overlooks the intrinsic interdependence between the two. Emotional expression in singing is frequently shaped by stylistic vocal techniques, while stylistic interpretation is itself influenced by perceived emotional tone. When these relationships are not modeled explicitly, existing systems are unable to exploit cross-task information that could enrich learned representations and enhance the fidelity of music style modeling.

In this study, we address the above challenge by proposing a multi-task learning framework that jointly performs emotion classification and singing style classification. The central idea is that explicitly modeling the correlation between the two tasks can enhance the performance of each task compared with training them independently. To enable effective cross-task knowledge exchange, we introduce a co-interactive relation module that allows the representations of one task to influence the other in a structured and adaptive manner. By jointly modeling singing style and emotional expression, the proposed framework captures richer dependencies in vocal signals, which leads to improved predictive performance and yields more interpretable latent representations.

To ensure the quality and reliability of the data used in our experiments, we constructed our dataset based on recordings captured with professional studio equipment. These devices function as acoustic sensors, enabling precise measurement of vocal timbre, dynamics, and other expressive nuances during singing. After recording, we employed state-of-the-art speech-to-text models to process and align the recordings, providing synchronized textual representations that complement the acoustic features. This multi-stage data preparation ensures that the proposed framework operates on clean, semantically aligned, and acoustically detailed input data.

Our main contributions are summarized as follows:

- **Joint modeling of correlated tasks.** We present a multi-task learning framework that performs emotion classification and singing style classification in a unified setting. By explicitly modeling the relationship between the two tasks, the framework achieves more accurate and coherent predictions than single-task baselines.
- **Broad applicability across model architectures.** We show that the advantages of joint training are not tied to a particular backbone network. Experiments conducted with Transformer, TextCNN, and BERT architectures consistently produce performance gains, demonstrating the generality and robustness of the proposed approach.
- **State-of-the-art performance and interpretability.** The proposed framework achieves the best results on our benchmark singing dataset under a singer-wise split. Further analysis illustrates how emotional expression and vocal style interact within the learned representations, offering interpretable insights that are valuable for both theoretical understanding and practical music applications.

## 2. Related Work

**Figure 1:**
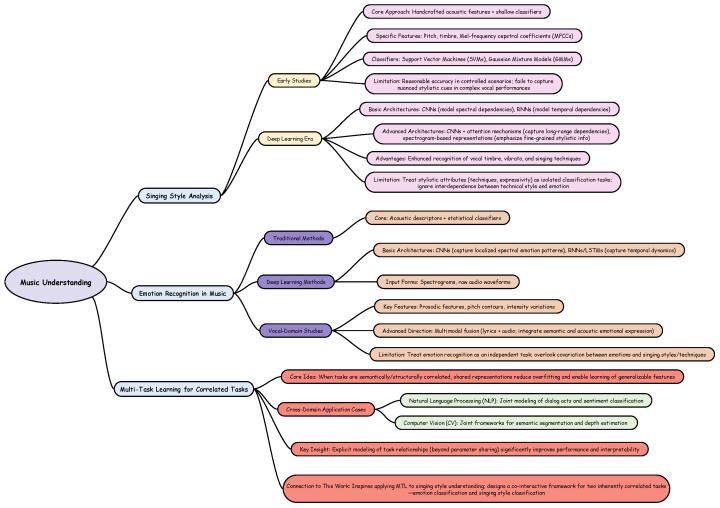
Overview of related research areas.

As illustrated in Figure 1, a substantial body of research has been conducted on music style analysis and music emotion recognition, and relevant progress has also been made in multi-task learning for correlated tasks. However, multi-task frameworks that specifically integrate singing style analysis with emotion recognition remain limited. Our work aims to address this gap by exploring their joint modeling.

### 2.1. Singing Style Analysis

Singing style analysis has long been recognized as an important topic in music information retrieval (MIR). Early research relied on handcrafted acoustic descriptors such as pitch, timbre, and Mel-frequency cepstral coefficients (MFCCs) to represent vocal characteristics [19]. These features were typically used together with shallow classifiers such as support vector machines or Gaussian mixture models to distinguish singers or stylistic tendencies [20]. While these approaches produced reasonable results in controlled scenarios, they often failed to capture the subtle stylistic cues present in expressive singing, especially when multiple vocal techniques co-occurred within complex musical interpretations [21].

With the development of deep learning, convolutional neural networks and recurrent neural networks became widely adopted in MIR research due to their enhanced capacity for modeling spectral and temporal properties of vocal signals [22]. CNN-based methods demonstrated effectiveness in representing vocal timbre and local harmonic structure [23], whereas RNN-based methods showed advantages in capturing temporal gestures such as vibrato and legato phrasing [24]. More recent work has explored hybrid architectures that combine convolutional feature extraction with attention mechanisms to capture longer-range contextual dependencies [25]. Spectrogram-based representations have also been used extensively to highlight fine-grained stylistic details in tone color and articulation [26].

Despite these advances, most research continues to treat stylistic properties such as vocal technique and expressive emotion as separate classification tasks. This fragmented approach limits the ability to model the interdependence between technical style and emotional expression, which jointly influence how singing style is perceived and interpreted [27].

### 2.2. Emotion Recognition in Music

Music emotion recognition (MER) is a central problem in music information retrieval that aims to automatically identify the emotional states conveyed in audio signals. Early studies relied on acoustic descriptors such as spectral centroid, tempo, and Mel-frequency coefficients, paired with statistical classifiers including support vector machines and Gaussian mixture models [28]. Although effective to a certain degree, these approaches struggled to represent the complex and nonlinear interactions among timbre, dynamics, and perceived emotional expression [29].

With the development of deep learning, researchers have increasingly adopted neural architectures capable of learning hierarchical and task-specific representations directly from spectrograms or raw waveforms [30]. Convolutional neural networks have shown strong performance in capturing local spectral cues associated with emotional intensity [23], while recurrent architectures such as LSTMs and GRUs better model temporal structures across musical phrases [31]. More recent work has explored hybrid and multimodal approaches that integrate audio, lyrics, and visual information to construct richer affective representations [32,33]. On the other hand, ref. [34] proposes a Spatio-Temporal Representation Fusion Learning Network (STRFLNet) that effectively integrates EEG features from both spatial and temporal dimensions for emotion recognition. Moreover, ref. [35] designs a light-weight residual convolution-based capsule network to achieve accurate EEG emotion recognition with a computationally efficient architecture.

In the vocal domain, prosodic cues such as pitch contour, energy fluctuation, and articulation play a central role in shaping emotional perception [36]. Recent studies further combine vocal acoustics with lyrical semantics to bridge the connection between expression and linguistic meaning [37]. However, most MER systems continue to treat emotion recognition as an isolated learning objective, without considering its interaction with stylistic vocal techniques. This separation limits their ability to capture the expressive hierarchy of vocal performance, where technique and emotion are inherently interdependent.

### 2.3. Multi-Task Learning for Correlated Tasks

Multi-task learning (MTL) offers a principled paradigm for jointly optimizing related tasks through shared representation learning [13,38]. This approach not only mitigates overfitting but also encourages the extraction of features that generalize more effectively across contexts. In natural language processing, for example, jointly modeling dialogue acts and sentiment has been shown to improve both predictive accuracy and interpretability [39,40]. In computer vision, related tasks such as semantic segmentation, depth estimation, and surface normal prediction benefit from shared feature spaces that reinforce common structural cues [41,42,43].

Recent work in MTL has moved beyond simple parameter sharing toward explicitly modeling relationships between tasks. Strategies such as dynamic loss weighting [44] and uncertainty-based balancing [41] adjust learning emphasis across tasks, while co-interactive or cross-attention mechanisms facilitate direct information exchange between task-specific representations [45]. These methods demonstrate that explicitly modeling task relationships can substantially enhance both performance and interpretability.

Motivated by these insights, our work applies the MTL paradigm to singing voice analysis, jointly modeling emotion classification and singing style classification. By capturing the correlations between expressive affect and vocal technique, we aim to improve the fidelity of learned representations and provide a more interpretable understanding of stylistic expression in vocal performance.

Traditional MTL frameworks typically rely on shared feature encoders or task-specific heads, where information flows implicitly through shared parameters. While effective, these architectures do not explicitly model the interaction between tasks such as singing style and emotion, and therefore cannot capture their fine-grained co-variation patterns. In contrast, our proposed bidirectional co-interactive relation module introduces a two-way information exchange mechanism that simultaneously allows the emotion branch to query style features and the style branch to query emotion features. This results in (1) explicit modeling of cross-task relations, (2) interpretable attention patterns that reveal how vocal style influences emotional perception and vice versa, and (3) enhanced generalization, as the module encourages the network to jointly reason over correlated acoustic cues.

## 3. Methods

### 3.1. Data Collection and Preprocessing

To ensure the high fidelity of the input data, we constructed a specialized dataset under strictly controlled acoustic conditions. The data acquisition pipeline consists of a high-resolution audio recording followed by automatic speech-to-text conversion.

**Figure 2:**
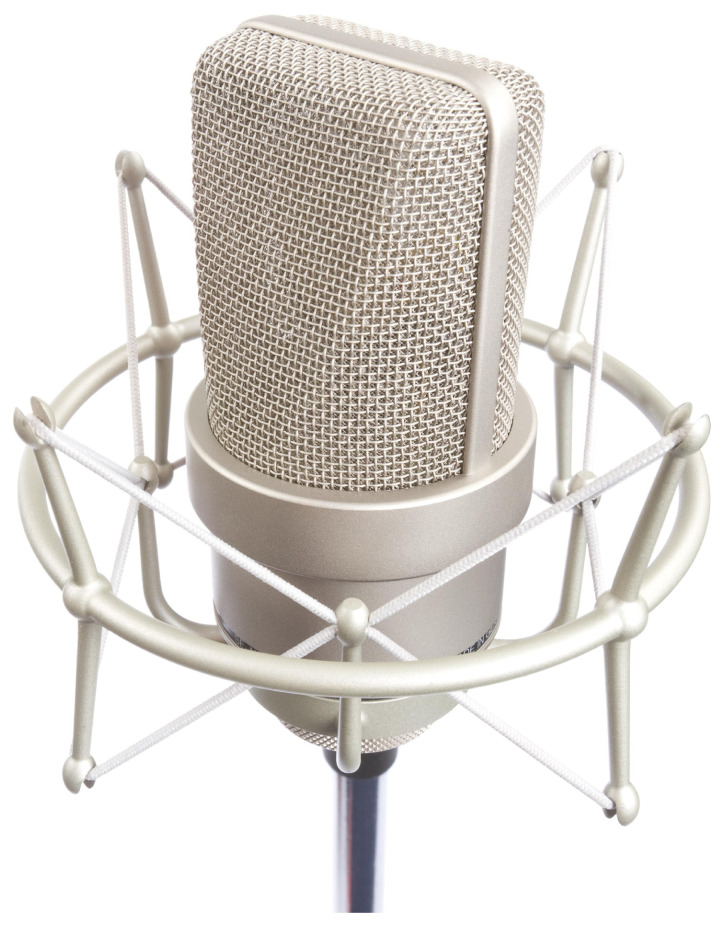
The professional audio recording setup used for data collection, including the Neumann TLM 103 microphone and high-fidelity audio interface.

The vocal recordings were collected in a professional recording studio environment, specifically designed to minimize reverberation and external noise. As shown in Figure 2, we utilized professional-grade condenser microphones (Neumann TLM 103 Manufacturer: Georg Neumann GmbH - City: Berlin - Country: Germany) connected via a high-fidelity audio interface to capture the vocal performances. Functioning as precision acoustic sensors, these devices allowed for the detailed measurement of vocal timbre and dynamic variations [21].

All audio signals were digitized at a sampling rate of 48 kHz with a 24-bit resolution. To standardize the signals, we applied fundamental signal processing steps, including DC-offset removal and amplitude normalization. Crucially, we refrained from applying aggressive denoising algorithms to preserve fine-grained expressive cues, such as breath sounds and vibrato, which are essential for stylistic analysis [19].

To obtain the textual content corresponding to the vocal performances, we processed the raw audio waveforms using the **Whisper** model (specifically the large-v3 architecture) [46], a state-of-the-art open-source automatic speech recognition (ASR) system. This automated pipeline effectively transcribes and aligns the sung lyrics with the audio segments [26]. By converting the acoustic signals into text sequences, this step provides the necessary linguistic information to complement the audio features for the subsequent analysis.

**Figure 3:**
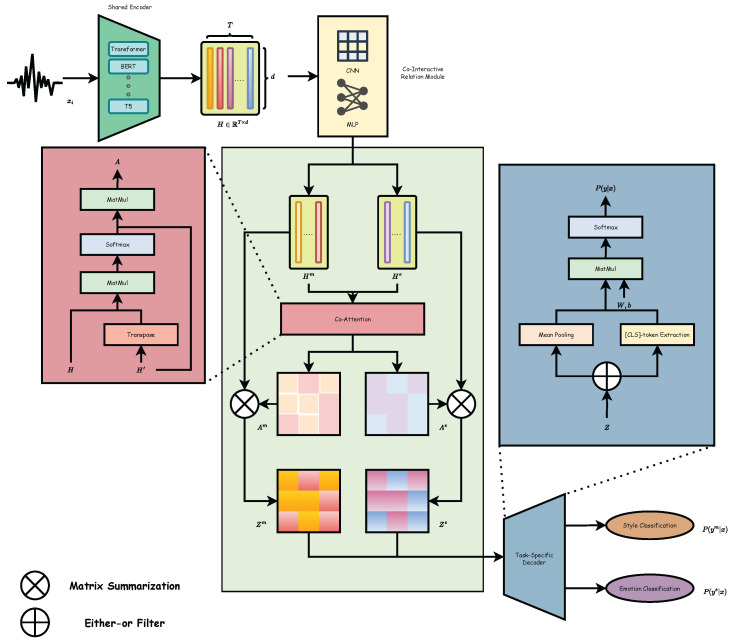
Overview of the proposed framework, which consists of three main components: a shared encoder, a co-interactive relation module, and two task-specific decoders. In the Co-Interactive Module: The *Either-or Filter* (Input/Output: $R^{B\times T\times d}$) employs a learned gating mask to selectively preserve task-specific information while filtering noise. The *Matrix Summarization* (Input: $R^{B\times T\times T}$, Output: $R^{B\times d}$) aggregates pairwise attention scores via pooling to form a compact global descriptor of cross-task correlations.

Figure 3 presents the overall architecture of the proposed framework. The following subsections describe the formulation and components of the model in detail.

### 3.2. Problem Formulation

We consider the joint task of singing style classification and emotion classification. Let $D={\left(x_{i},y_{i}^{m},y_{i}^{e}\right)}_{i=1}^{N}$ denote the dataset, where $x_{i}$ is an input vocal segment, $y_{i}^{m}$ is the singing style label (for example, Bel canto or Pop), and $y_{i}^{e}$ is the emotion label (for example, joyful or sad). The goal is to learn a model f(x;θ) parameterized by θ, which predicts both $y^{m}$ and $y^{e}$ for a given input.

Unlike single-task settings that treat the two tasks independently, we aim to make use of their correlation. The joint learning objective can be written as:

(1) $$L=\sum_{i=1}^{N}\left(\log P\left(y_{i}^{m}|x_{i};\theta \right)+\log P\left(y_{i}^{e}|x_{i};\theta \right)\right),$$

where both predictions are informed by shared and interactive representations.

### 3.3. Shared Encoder

Each input $x_{i}$ is represented as either a sequence of acoustic features (for example, mel-spectrograms) or token-level waveform embeddings. A pre-trained encoder backbone such as Transformer, BERT, or T5 is employed to learn contextualized sequence representations:

(2) $$H=\mathrm{Encoder}\left(x\right)\in R^{T\times d},$$

where *T* is the sequence length and *d* is the hidden dimension. The encoder captures both local acoustic variations and long-range contextual dependencies, forming a shared feature space for both tasks.

### 3.4. Co-Interactive Relation Module

Given the shared representation $H\in R^{T\times d}$, we first obtain task-specific features through lightweight projection layers:

(3) $$\begin{array}{cc} H^{m} & =f_{m}\left(H\right)\in R^{T\times d}, \end{array}$$

(4) $$\begin{array}{cc} H^{e} & =f_{e}\left(H\right)\in R^{T\times d}. \end{array}$$

#### 3.4.1. Bidirectional Co-Attention

To explicitly model cross-task interaction, we construct independent query–key–value projections for the two tasks:

(5) $$\begin{array}{c} Q^{m}=H^{m}W_{Q}^{m},\;K^{e}=H^{e}W_{K}^{e},\;V^{e}=H^{e}W_{V}^{e}, \end{array}$$

(6) $$\begin{array}{c} Q^{e}=H^{e}W_{Q}^{e},\;K^{m}=H^{m}W_{K}^{m},\;V^{m}=H^{m}W_{V}^{m}, \end{array}$$

where all projection matrices are in $R^{d\times d}$. Thus, the cross-attention maps become

(7) $$\begin{array}{cc} A^{m} & =\mathrm{softmax}\;\left(\frac{Q^{m}{\left(K^{e}\right)}^{⊤}}{\sqrt{d}}\right)V^{e}\;\;\in R^{T\times d}, \end{array}$$

(8) $$\begin{array}{cc} A^{e} & =\mathrm{softmax}\;\left(\frac{Q^{e}{\left(K^{m}\right)}^{⊤}}{\sqrt{d}}\right)V^{m}\;\;\in R^{T\times d}. \end{array}$$

#### 3.4.2. Gated Residual Integration

To avoid one task overwhelming the other, we introduce task-specific gating factors:

(9) $$\begin{array}{c} g_{m}=\sigma \left(W_{g}^{m}H^{m}\right),\;g_{e}=\sigma \left(W_{g}^{e}H^{e}\right), \end{array}$$

where $g_{m},g_{e}\in R^{T\times d}$. The final fused representations are then

(10) $$\begin{array}{cc} Z^{m} & =H^{m}+g_{m}⊙A^{m}, \end{array}$$

(11) $$\begin{array}{cc} Z^{e} & =H^{e}+g_{e}⊙A^{e}. \end{array}$$

This gating ensures that each task selectively controls how much information it absorbs from the other branch, preventing dominance while preserving task-specific structure.

Either-or Filter selectively preserves task-specific information by applying a learned gating mask $g\in {\left[0,1\right]}^{d}$ to each feature channel. Given an input representation h, the filter outputs $h^{\prime }=g⊙h+\left(1-g\right)⊙\tilde{h}$, where $\tilde{h}$ denotes an alternative transformed feature. This mechanism prevents one task from dominating the shared representation and allows conditional routing of information.

Matrix Summarization aggregates the pairwise attention responses into a compact representation by applying row-wise and column-wise pooling to the attention matrix. This summarizes cross-task alignment patterns into a fixed-size descriptor that can be fed into the subsequent fusion layers.

This module allows the model to capture cross-task cues, such as how particular vocal techniques correlate with specific emotional expressions.

#### 3.4.3. Interpretation of Task Interaction

The bidirectional co-interactive module can be viewed as aligning correlated subspaces between the two tasks. Emotion and style features often share consistent spectral cues—such as changes in harmonic energy distribution, resonance structure, and temporal intensity variations. Formally, the cross-attention matrices select dimensions of $H^{m}$ and $H^{e}$ that exhibit correlated patterns, enabling mutual refinement while preserving task-specific components through gated residual connections.

### 3.5. Task-Specific Decoders

The refined representations are passed through lightweight classification heads. For singing style classification:

(12) $$P\left(y^{m}|x\right)=\mathrm{softmax}\left(W_{m}\cdot \mathrm{Pool}\left(Z^{m}\right)+b_{m}\right),$$

and for emotion classification:

(13) $$P\left(y^{e}|x\right)=\mathrm{softmax}\left(W_{e}\cdot \mathrm{Pool}\left(Z^{e}\right)+b_{e}\right),$$

where Pool(·) denotes either mean pooling or [CLS]-token extraction.

The decoders are deliberately kept simple so that the burden of modeling cross-task dependencies rests primarily on the encoder and co-interactive module, enhancing interpretability.

### 3.6. Joint Optimization

The final objective of our framework is to optimize both singing style and emotion classification in a unified manner. To this end, we define the overall training loss as a weighted sum of the two task-specific losses:

(14) $$L=\lambda_{m}L_{m}+\lambda_{e}L_{e},$$

where $L_{m}$ and $L_{e}$ denote the classification losses for singing method and emotion, respectively, and $\lambda_{m},\;\lambda_{e}\in R^{+}$ are non-negative coefficients that control the relative importance of each task.

For categorical prediction, we adopt the standard cross-entropy loss:

(15) $$\begin{array}{cc} L_{m} & =-\sum_{i=1}^{N}\sum_{c=1}^{C_{m}}1\left[y_{i}^{m}=c\right]\cdot \log P\left(y_{i}^{m}=c|x_{i}\right), \end{array}$$

(16) $$\begin{array}{cc} L_{e} & =-\sum_{i=1}^{N}\sum_{c=1}^{C_{e}}1\left[y_{i}^{e}=c\right]\cdot \log P\left(y_{i}^{e}=c|x_{i}\right), \end{array}$$

where $C_{m}$ and $C_{e}$ are the number of categories for method and emotion classification, and 1[·] is the indicator function.

**Balancing Task Weights**: In our main experiments, we set $\lambda_{m}=\lambda_{e}=1$ to give equal weight to both tasks. However, this formulation is flexible:

- If one task is more difficult (e.g., emotion classification), assigning a larger $\lambda_{e}$ biases the model to prioritize improvements in that task.
- Conversely, reducing $\lambda_{m}$ can prevent over-dominance of the easier singing method classification task, thereby encouraging more balanced gradient updates.

**Dynamic Weighting Strategies**: Beyond fixed weights, the prior multi-task learning literature has shown the benefits of dynamic task balancing. One example is the uncertainty-based weighting method [41], which automatically adjusts $\lambda_{m}$ and $\lambda_{e}$ based on task-dependent homoscedastic uncertainty:

(17) $$L=\frac{1}{2\sigma_{m}^{2}}L_{m}+\frac{1}{2\sigma_{e}^{2}}L_{e}+\log\sigma_{m}\sigma_{e},$$

where $\sigma_{m}$ and $\sigma_{e}$ are learned parameters. This formulation adaptively balances tasks as training progresses.

### 3.7. Training and Inference

During training, both tasks are optimized jointly using backpropagation. At inference time, the model outputs both singing style and emotion predictions for a given input segment. This joint design ensures that each task benefits from the other: style predictions are enriched by emotion-aware signals, and emotion predictions are grounded in stylistic cues. Consequently, the framework not only improves quantitative accuracy but also provides interpretable insights into the interplay between vocal technique and emotional expression.

### 3.8. Potential Extensions: Adaptive Loss Weighting

Uncertainty-based task weighting is a promising direction for future improvement. Although we adopt fixed task weights in all experiments for stability—due to the heterogeneous entropy of the two tasks and the strong label imbalance in our dataset—adaptive weighting may further enhance robustness in settings where both tasks exhibit comparable uncertainty. Integrating such loss-weighting strategies with our co-interactive module represents an interesting direction for future exploration.

## 4. Experiments and Results

### 4.1. Correlation Analysis of Labels

Before presenting the main experimental results, we first analyze the correlation between the two types of labels in our dataset, namely *singing method* and *emotion*. This analysis provides empirical evidence that these tasks are not independent, but instead exhibit structured relationships that can be exploited by a multi-task framework.

Specifically, we construct a co-occurrence matrix $M\in R^{C_{m}\times C_{e}}$, where $M_{ij}$ counts how many times singing method *i* co-occurs with emotion *j* in the dataset. From this matrix, we derive normalized conditional probabilities:

(18) $$P\left(y^{e}|y^{m}\right)=\frac{M_{ij}}{\sum_{j}M_{ij}},\;P\left(y^{m}|y^{e}\right)=\frac{M_{ij}}{\sum_{i}M_{ij}},$$

which reveal how strongly each singing method is associated with specific emotions, and vice versa.

Our analysis shows clear patterns of dependency. For instance, *Pop* is predominantly associated with high-arousal emotions such as *joyful* and *energetic*, while *Bel canto* appears more frequently with *sad* or *soft* emotions. These correlations suggest that emotions are often conveyed through specific vocal techniques, and that recognizing one can provide informative cues for predicting the other.

As shown in Table 1, this empirical evidence supports our central hypothesis: since singing method and emotion classification are correlated, training them jointly allows the model to capture their shared structure and improves performance compared to learning them in isolation.

### 4.2. Experimental Setup

The experimental dataset was constructed from high-fidelity singing recordings collected using professional recording devices. These devices, functioning as precision acoustic sensors, captured fine-grained details of vocal performance under controlled studio conditions. To further refine the dataset, each recording was processed and aligned with its corresponding lyrics using pre-trained speech-to-text alignment models, ensuring accurate synchronization between the audio and textual modalities.

To evaluate the effectiveness of our proposed multi-task learning framework, we conducted experiments on a benchmark singing dataset annotated with both singing style labels (e.g., Pop, Bel canto) and emotion labels (e.g., happy, sad).

**Data Split.** The dataset was randomly divided into training (70%), validation (10%), and testing (20%) sets. To reduce the influence of data imbalance, we adopted stratified sampling so that the distribution of labels remained consistent across subsets.

**Baselines.** We compared three training settings:

- **Multi-task**: Jointly training the model on both singing style classification and emotion classification.
- **Singing-style-only**: Training the model solely for singing style classification.
- **Emotion-only**: Training the model solely for emotion classification.

**Backbone Models.**

**BERT-base** [47], an encoder-only model, excels in tasks that require deep understanding of the input text. Its bidirectional training mechanism allows it to capture contextual nuances effectively, making it ideal for tasks such as sentiment analysis and question answering. However, BERT’s lack of a decoder component limits its utility in tasks that involve text generation. Additionally, its computational demands can be substantial, particularly when fine-tuning for specific tasks.

**GPT2-small** [48], a decoder-only model, is designed for text generation tasks. Its autoregressive nature enables it to generate coherent and contextually relevant text based on the input prompt. This model is particularly useful for tasks like text continuation, summarization, and dialogue systems. However, GPT2’s unidirectionality means it can only consider past context, potentially limiting its comprehension capabilities compared to bidirectional models.

**T5-small** [49], an encoder–decoder model, combines the strengths of both paradigms, offering a versatile approach to NLP tasks. It can effectively process input text through the encoder and generate output through the decoder, making it suitable for a wide array of tasks, including translation, summarization, and question answering. T5’s unified framework simplifies the process of adapting the model to different text-to-text tasks, although it may require more computational resources and training data compared to single-purpose models.

**Implementation Details.** Models were trained with the Adam optimizer (learning rate = 1 × 10^−4^, batch size = 16). Early stopping was applied based on validation accuracy to prevent overfitting. All experiments were repeated three times, and the average results are reported.

**Evaluation Metrics.** Accuracy was used as the evaluation metric for both tasks. Additionally, we report relative improvements in multi-task settings to better quantify the benefit of leveraging task correlations.

### 4.3. Dataset and Annotation Details

The recordings used in this study were captured in a controlled, acoustically treated studio environment using professional-grade condenser microphones (Neumann TLM 103) connected via an audio interface. Audio was sampled at **48 kHz** with **24-bit** resolution. Preprocessing included DC-offset removal and amplitude normalization; no aggressive denoising was applied to preserve expressive acoustic cues.

Segment-level labels for singing style and emotion were obtained by human annotation as follows: each sample was independently labeled by two trained annotators (background in musicology or vocal studies). In cases of disagreement, a third expert adjudicator determined the final label.

#### Data Splitting and Leakage Prevention

Since all recordings in our dataset were collected from our own controlled recording pipeline, we carefully ensured that no audio from the same singer or the same recording session appears across the training, validation, and test splits.

All clips belonging to the same singer are placed entirely into one split, preventing potential information leakage caused by shared timbre, recording environment, or singing habits. This stricter split strategy avoids optimistic evaluation bias and provides a more reliable estimate of real-world generalization.

### 4.4. Datasets Analysis

**Figure 4:**
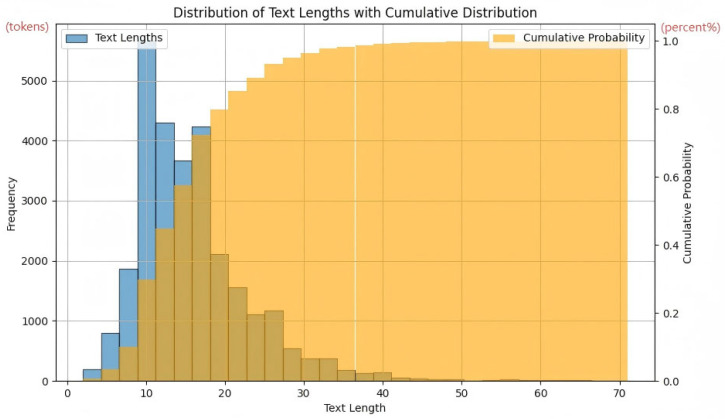
The figure shows the distribution of music text lengths, revealing that the majority of lyrics fall within the 10–20 word range.

Figure 4 illustrates the frequency distribution of text lengths and their cumulative distribution in the music lyric dataset. It can be observed from the chart that the frequency distribution of text lengths exhibits a pronounced right-skewed characteristic, with most lyrics being relatively short and concentrated between 10 and 30 words. Specifically, approximately 80% of the lyrics are no longer than 30 words, a finding further validated by the cumulative distribution curve. The cumulative distribution curve rises sharply within the 10 to 30-word range, indicating that lyrics within this length interval make up the majority of the dataset. Furthermore, despite the majority of lyrics being short, there are a few that exceed 50 words, even approaching 70 words. These longer texts may represent special types of lyrics, such as songs with narrative strength or lyrics containing repeated sections. Although these longer texts are a small proportion of the dataset, they may have certain impacts on music text analysis and model training.

The cumulative distribution curve further reveals the distribution characteristics of lyric lengths. As shown in Figure 4, the cumulative distribution curve rises sharply within the 10- to 30-word range, indicating that lyrics within this length interval constitute the majority of the dataset. As the length of the lyrics increases, the rate of increase in cumulative probability slows down, eventually leveling off around 70 words, approaching 1.

This result indicates that most lyrics are relatively short and the length distribution exhibits a pronounced right-skewed characteristic. This distribution characteristic may be related to the creative habits and expression methods of music lyrics, which typically need to convey emotions and stories within a limited space, thus tending to use concise and condensed language.

In summary, the analysis of the text length distribution in the music lyric dataset reveals that most lyrics are relatively short and exhibit a pronounced right-skewed length distribution. These findings provide an important reference for music text analysis and model selection and offer foundational data support for subsequent research. Future studies can further explore the relationship between lyric length and factors such as music style and emotional expression. We performed a statistical analysis of the emotion categories and singing methods to better understand the distribution of categories in the dataset. The results for each category are presented below.

#### 4.4.1. Emotion Category Statistics

**Figure 5:**
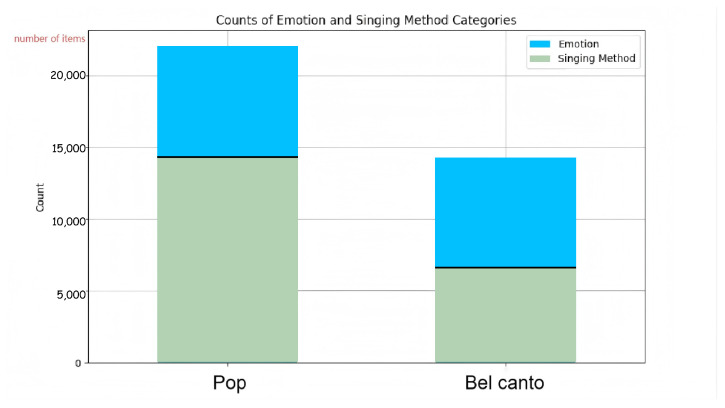
The figure illustrates the distribution of emotions across different music genres. It can be observed that both Pop and El Canto encompass a wide range of emotional categories, indicating that the dataset exhibits a reasonable and balanced distribution.

Table 2 and Figure 5 show the distribution of different emotion categories in the dataset. As can be seen, happy and sad are the predominant emotion categories, with 14,321 and 14,259 samples, respectively. In contrast, the categories happy-sad-mixed have very few samples, with only 48 instances each, and can be considered negligible.

#### 4.4.2. Singing Method Statistics

Table 3 shows the distribution of different singing methods. Regarding singing methods, Pop is the most common type, accounting for the vast majority of the dataset with 22,055 samples, which is approximately 76.2%. In contrast, Bel canto has 6573 samples, which accounts for around 23.8%.

From the statistical analysis above, it is clear that happy and sad are the dominant emotion categories in the dataset, together constituting the vast majority of the data. In contrast, the happy–sad–mixed categories are very rare, which warrants further examination to determine whether these categories are valid or potentially noise in the data. In terms of singing methods, Pop clearly dominates the dataset with a sample count of 22,055, which represents approximately 76.2% of the data. This indicates that Pop music is the most prevalent singing style in this dataset. On the other hand, Bel canto has a relatively small sample size, suggesting that it is less common in the dataset. Further investigation may be needed to explore the reasons behind this.

This section analyzed the distribution of emotion categories and singing methods. Future research could delve deeper into the relationships between these categories, preferences for singing styles, and their correlations with other variables such as audio features, singer characteristics, and so on.

### 4.5. Results

Table 4 summarizes the experimental results. Overall, the findings indicate that multi-task learning consistently outperforms single-task baselines across all tested models, particularly for emotion classification, confirming our hypothesis that leveraging task correlations benefits both tasks.

For singing style classification, the performance of the multi-task setting is slightly higher than or comparable to the singing-style-only baseline, suggesting that auxiliary emotion information does not harm and may even refine the recognition of singing techniques. The improvements are especially evident for emotion classification: in T5-small, accuracy increases from 0.3410 (emotion-only) to 0.3551 (multi-task); in GPT2-small, from 0.8160 to 0.8410; and in BERT, from 0.8768 to 0.8795. These consistent gains highlight the robustness of our approach across architectures.

Among the tested backbones, BERT achieves the best absolute performance, reaching 0.9987 accuracy on singing style classification and 0.8795 on emotion classification under the multi-task setting. This shows that our framework can effectively generalize across different architectures while still maintaining strong task-specific performance.

For fairness, we also tested a class-weighted cross-entropy variant of the single-task emotion classifier (e.g., BERT fine-tuned only for emotion). The performance differed from the original single-task baseline by only ±0.3–0.5% in accuracy and macro-F1, and the multi-task model still consistently outperformed both versions. We therefore keep the main tables focused on the standard setting for simplicity.

### 4.6. Ablation Study

To validate the effectiveness of each component in our framework, we performed an ablation study on the BERT-base backbone. Specifically, we evaluated three model variants:

- **w/o Co-Interactive Module**: Removing the co-interactive relation module, i.e., tasks share only the encoder without explicit cross-task interactions.
- **Single-task**: Training two independent models for singing style and emotion classification, respectively.
- **Full Model**: Our proposed joint framework with co-interactive relation module.

As shown in Table 5, removing the co-interactive relation module leads to a significant drop in emotion classification accuracy (from 0.8795 to 0.8613), confirming that explicit cross-task modeling is essential. Similarly, compared to single-task baselines, our full model consistently improves both singing style and emotion recognition, demonstrating the mutual benefits of multi-task learning.

To better understand the effectiveness of the proposed co-interactive relation module, we further compare it against two alternative task-interaction strategies commonly used in multi-task learning: Single-direction cross-attention, where the singing-style representation serves as a query to attend to the emotion features (style→emotion), enabling only one-way information flow. Concatenate, a widely used feature-level fusion baseline, where the concatenated representations are processed by a multilayer perceptron without explicit attention modeling.

Table 6 reports the comparison results. Our bidirectional co-attention achieves the highest emotion macro-F1 while maintaining competitive singing-style accuracy. The improvements indicate that allowing the two tasks to mutually query each other provides richer cross-task cues than single-direction attention or simple concatenation. These findings support the empirical justification for our design and demonstrate that bidirectional cross-task reasoning is essential for enhancing multi-task vocal understanding.

### 4.7. Analysis Under Class Imbalance

To more comprehensively evaluate the robustness of our model under challenging data distributions, we further analyze its behavior on underrepresented categories and imbalanced label conditions. To better understand the effect of label imbalance, we experiment with common imbalance mitigation strategies: (1) baseline training without correction, (2) class-weighted cross entropy, and (3) oversampling of minority classes. Table 7 shows that class-weighted cross-entropy yields the most consistent improvements in rare-class recall while maintaining overall performance. Oversampling can benefit extremely rare categories but risks overfitting.

These analyses collectively demonstrate that while the proposed model already performs strongly, imbalance-aware training further improves robustness on underrepresented emotion–style combinations.

## 5. Discussion

The experimental results, ablation analysis, and case studies all point to a consistent conclusion: multi-task learning provides complementary signals that enhance both singing style and emotion classification. Importantly, the framework is architecture-agnostic, working effectively across encoder-only (BERT), decoder-only (GPT2), and encoder–decoder (T5) models. This suggests that the proposed framework can be flexibly applied to a variety of backbone models and potentially extended to other music-related classification tasks.

### 5.1. Characteristics of Vocal Styles Are Not Clearly Defined

In the lyrics, specific vocal styles (such as Pop and Bel canto) may not be directly reflected in the text itself, especially for a text-based model. Even if the lyrics align with a certain vocal style, the model may not have enough information to distinguish the subtle differences between these styles. For instance, the “Bel canto” vocal style is typically associated with highly technical singing and classical music, while the “Pop” vocal style is more modern and casual. The model likely relies more on the structure of the lyrics rather than the tonal qualities and techniques inherent in the vocal style. This leads to incorrect judgments when audio information is absent.

### 5.2. Lack of Context in Lyrics

In both of the error samples, the lyrics do not provide sufficient contextual information, making it difficult for the model to accurately classify the style based on the text alone. The lyrics may be too brief or abstract, failing to convey clear stylistic traits. The model may not have effectively considered context, emotional nuances, or specific artistic expressions (such as the strength of emotion or rhythmic elements) in making its predictions, leading to incorrect classifications.

### 5.3. Model’s Bias Toward Certain Styles

Based on the incorrect predictions, it seems that the model tends to overestimate the likelihood of complex or ambiguous lyrics being classified as “Bel canto” rather than “Pop”. This could be due to the fact that the “Bel canto” style is often associated with more intricate or artistic performances, while the “Pop” style tends to be more straightforward and simple. The model might lack sufficient “discriminative” ability to differentiate between subtle stylistic differences, especially when the lyrics appear ambiguous. In such cases, the model is more likely to default to the style it perceives as more complex or artistic.

### 5.4. Impact of Pre-Training

As shown in Table 8, the drop from 86.1% to 84.2% after pre-training contradicts the common belief that pre-training always helps. The 1.9-point decrease, however, exposes the unique nature of lyric sentiment classification. Lyrics are highly colloquial, packed with repeated choruses and sharp emotional polarity—distributions that diverge markedly from the news and encyclopedia domains on which the encoder was pre-trained. After fine-tuning, the model over-associates frequent, generic affective tokens (“love”, “baby”) and overlooks music-specific metaphors, irony, and rhythmic cues, yielding negative transfer. Thus, while pre-training enlarges the model’s capacity for “general sentiment,” it blunts its sensitivity to the subtle, domain-specific emotions expressed in songs. The evidence argues for either training from scratch on in-domain data or adding a second-stage “lyric-only” pre-training step to unlock the true benefit of transfer learning for musical sentiment analysis.

### 5.5. Error Analysis

Although our proposed multi-task framework achieves consistent improvements across both tasks, several sources of errors remain. We conducted an in-depth analysis of the misclassified samples in the test set and summarized the major observations as follows.

First, for emotion classification, most errors occur in cases where the emotional expression is subtle or ambiguous. For example, performances labeled as *neutral* are sometimes predicted as *melancholic* due to low vocal intensity, or confused with *joyful* when the rhythm is relatively fast. This suggests that emotion recognition is inherently subjective, and additional contextual cues (e.g., lyrics or performance context) may be needed to further disambiguate.

Second, for singing style classification, confusions primarily happen between acoustically similar techniques. A typical case is the misclassification of *belting* as *vibrato* when the vibrational frequency overlaps with high-intensity pitch variations. Similarly, *falsetto* and *head voice* can be easily confused in boundary cases, reflecting the fine-grained nature of style distinctions.

Third, we observed that data imbalance also contributes to errors. Styles such as *belting* and emotions like *joyful* are more frequent in the dataset, leading to relatively better performance, whereas rare categories (e.g., *melancholic vibrato*) exhibit higher error rates.

Overall, these findings indicate that while the multi-task framework provides mutual benefits, there remain challenges related to label subjectivity, acoustic similarity, and data imbalance. Addressing these issues by incorporating richer contextual information, designing more discriminative representations, and exploring data augmentation techniques are promising directions for future work.

## 6. Conclusions

In this work, we investigated the task of singing style understanding by focusing on two highly correlated subtasks: singing style classification and emotion classification. We proposed a multi-task learning framework that jointly models these tasks through a shared encoder, a co-interactive relation module, and task-specific decoders. Our experiments across multiple backbone architectures, including T5, GPT2, and BERT, demonstrated that joint training consistently outperforms single-task baselines. In particular, we observed notable improvements in emotion classification, confirming that explicitly leveraging task correlations can enhance performance and interpretability.

Beyond achieving state-of-the-art results on a benchmark dataset, our framework provides new insights into the relationship between vocal techniques and emotional expression, offering practical value for applications such as music information retrieval, personalized recommendation, and AI-assisted music creation. In future work, we plan to extend our framework to multimodal settings by incorporating lyrical and visual information and to explore fine-grained, continuous representations of singing styles for richer music understanding.

## Figures and Tables

**Table 1:** Co-occurrence statistics between singing method and emotion labels, along with the Inter-Annotator Agreement (IAA) reliability checks. Values indicate normalized conditional probabilities $P(y^{e}|y^{m})$ and Cohen’s κ coefficients.

Category/Label	IAA (Cohen’s κ)	Happy	Sad
Pop (Singing Method)	0.82	0.72	0.28
Bel canto (Singing Method)	0.86	0.35	0.65
Emotion Labels (Avg.)	0.79	-	

**Table 2:** Emotion Category Distribution.

Emotion Category	Count	Percentage (%)
happy	14,321	49.99
sad	14,259	49.98
happy-sad-mixed	48	0.16

**Table 3:** Singing Method Distribution.

Singing Method	Count	Percentage (%)
Pop	22,055	76.2
Bel canto	6573	23.8

**Table 4:** Comparison of classification accuracy and Macro-F1 scores under different training settings (mean ± std over 3 runs). The inclusion of Macro-F1 provides a more robust evaluation under class imbalance. Multi-task consistently improves performance over single-task baselines.

Model	Setting	Singing Style		Emotion	
		**Acc.**	**Macro-F1**	**Acc.**	**Macro-F1**
T5-small	Multi-task	0.9860 ± 0.0009	0.9652 ± 0.0015	0.3551 ± 0.0048	0.2840 ± 0.0051
	Singing-style-only	0.9759 ± 0.0012	0.9510 ± 0.0018	–	–
	Emotion-only	–	–	0.3410 ± 0.0053	0.2615 ± 0.0062
GPT2-small	Multi-task	0.9985 ± 0.0001	0.9960 ± 0.0002	0.8410 ± 0.0031	0.6120 ± 0.0045
	Singing-style-only	0.9979 ± 0.0002	0.9945 ± 0.0003	–	–
	Emotion-only	–	–	0.8160 ± 0.0034	0.5840 ± 0.0050
BERT-base	Multi-task	0.9987 ± 0.0001	0.9972 ± 0.0001	0.8795 ± 0.0024	0.6705 ± 0.0032
	Singing-style-only	0.9980 ± 0.0002	0.9958 ± 0.0002	–	–
	Emotion-only	–	–	0.8768 ± 0.0026	0.6610 ± 0.0038

**Table 5:** Ablation study on BERT-base backbone (mean ± std over 3 runs). The co-interactive module provides clear gains, especially for emotion classification.

Model Variant	Style Acc.	Emotion Acc.
Single-task	0.9980 ± 0.00015	0.8768 ± 0.0026
w/o Co-Interactive	0.9981 ± 0.00012	0.8613 ± 0.0031
Full Model	0.9987 ± 0.00010	0.8795 ± 0.0024

**Table 6:** Comparison of different task-interaction mechanisms on BERT-base backbone.

Interaction Mechanism	Style Acc	Emotion Acc
Single-direction cross-attn	0.9983	0.8690
Concatenate	0.9982	0.8662
**Bidirectional co-attention (ours)**	**0.9987**	**0.8795**

**Table 7:** Effect of imbalance-handling strategies on Style Acc, Emotion Acc, Emotion Macro-F1 and Rare-Class Recall.

Strategy	Style Acc	Emotion Acc	Emotion Macro-F1	Rare-Class Recall
Baseline	0.9987	0.8795	0.670	0.125
Class-weighted CE	0.9985	0.8760	0.725	0.360
Oversampling	0.9978	0.8730	0.705	0.300

**Table 8:** Comparison of Emotion Acc. with and without pretraining.

Condition	Emotion Acc.
No Pretrain	0.861
Pretrain	0.842

## Data Availability

The raw data supporting the conclusions of this article will be made available by the authors on request.
